# MetQ of Neisseria gonorrhoeae Is a Surface-Expressed Antigen That Elicits Bactericidal and Functional Blocking Antibodies

**DOI:** 10.1128/IAI.00898-16

**Published:** 2017-01-26

**Authors:** Evgeny A. Semchenko, Christopher J. Day, Kate L. Seib

**Affiliations:** Institute for Glycomics, Griffith University, Gold Coast, QLD, Australia; University of Texas at Austin

**Keywords:** ABC transporters, MetQ, Neisseria gonorrhoeae, adhesins, gonorrhea, methionine, vaccine candidate

## Abstract

Neisseria gonorrhoeae, the causative agent of the sexually transmitted infection (STI) gonorrhea, is a growing public health threat for which a vaccine is urgently needed. We characterized the functional role of the gonococcal MetQ protein, which is the methionine binding component of an ABC transporter system, and assessed its potential as a candidate antigen for inclusion in a gonococcal vaccine. MetQ has been found to be highly conserved in all strains investigated to date, it is localized on the bacterial surface, and it binds l-methionine with a high affinity. MetQ is also involved in gonococcal adherence to cervical epithelial cells. Mutants lacking MetQ have impaired survival in human monocytes, macrophages, and serum. Furthermore, antibodies raised against MetQ are bactericidal and are able to block gonococcal adherence to epithelial cells. These data suggest that MetQ elicits both bactericidal and functional blocking antibodies and is a valid candidate antigen for additional investigation and possible inclusion in a vaccine for prevention of gonorrhea.

## INTRODUCTION

Neisseria gonorrhoeae, the causative agent of the sexually transmitted infection (STI) gonorrhea, is a growing global health concern for which a vaccine is urgently needed (1, 2). It is estimated that there are more than 108 million cases of gonorrhea worldwide each year (1), resulting in a range of clinical outcomes, including severe sequelae. Recent reports of the emergence of multidrug-resistant strains highlight the need for global attention to address this issue (3), and the U.S. Centers for Disease Control and Prevention (CDC) has prioritized N. gonorrhoeae as one of three bacteria that pose an urgent public health threat for which immediate aggressive action is needed (4).

In women, N. gonorrhoeae infections of the lower genital tract (i.e., gonococcal cervicitis) remain asymptomatic in 50 to 80% of cases (5–7). If the infection is left untreated, gonococcal ascension from the cervix to the fallopian tubes occurs in up to 45% of women, which can result in pelvic inflammatory disease (PID; inflammation of the uterus, fallopian tubes, and/or ovaries) (8) and other sequelae that include spontaneous abortion, stillbirth, ectopic pregnancy, ophthalmia neonatorum, infertility, and disseminated infection (7). In men, gonococcal infection typically results in a symptomatic, localized inflammatory response of the urethra (i.e., urethritis). However, a proportion of infected men are asymptomatic (usually reported at rates of 1 to 3% [7, 9] but possibly as high as 30 to 40% [5, 6), and sequelae can include urethral stricture, urogenital tract abscesses, prostatitis, epididymo-orchitis, and infertility (7). Infection with N. gonorrhoeae also increases the rates of HIV replication, transmission, and infection in both men and women (10).

Vaccination is considered to be the best approach to reduce the transmission of communicable diseases; however, vaccine development has been challenging for N. gonorrhoeae, largely due to high rates of phase and antigenic variation, various mechanisms of immune evasion, and the lack of an animal model that accurately mimics disease transmission and progression (reviewed in reference 11). Investigation of the major outer membrane structures has generally been unsuccessful, with a series of human infection studies highlighting their variable nature. For example, gonococci expressing multiple variants of pilin (12, 13), lipooligosaccharide (LOS), and opacity-associated outer membrane proteins (Opa) were recovered from human male volunteers shortly after infection with an inoculum that was predominantly antigenically homogeneous (14). Consequently, the pilus-based vaccines tested in the 1990s provided no protection (15). Nevertheless, several potential vaccine targets have been identified, including the nitrite reductase, AniA; phospholipase D (PLD); transferrin-binding proteins, TbpAB; MtrE of the Mtr efflux pump complex; the outer membrane porin PorB; as well as the conserved 2C7 epitope of LOS (reviewed in references 11 and 16). Furthermore, genomics- and proteomics-based approaches (17, 18) have revealed other potential vaccine candidates, and the search for novel targets is ongoing.

ATP-binding cassette (ABC) transporters are highly conserved and play an important role in bacterial survival and pathogenicity (19). Consequently, they have been investigated as potential vaccine candidates for several bacteria, including Moraxella catarrhalis (20) and Streptococcus pneumoniae (21). ABC transporters consist of substrate binding, ATPase, and permease activities, which mediate the ATP-dependent transport of organic and inorganic molecules across cellular membranes (22). Many ABC transporters are localized in the cytoplasmic membrane; however, the substrate binding components have also been shown to be surface exposed in several pathogenic bacteria, where they may have additional functions, including adherence (23–25). A putative methionine binding component of an ABC transporter, genome-derived *N*eisseria antigen 1946 (GNA1946; also named NMB1946), was identified to be a potential candidate vaccine against Neisseria meningitidis (26), which is closely related to N. gonorrhoeae. The meningococcal GNA1946 is a lipoprotein that has a high level of sequence conservation between strains (>98% identity across its 287 amino acids) (26, 27) and is surface expressed and exposed (26), and mouse antiserum raised against GNA1946 showed complement-mediated bactericidal activity against heterologous N. meningitidis strains (26). Crystal structure analysis revealed that GNA1946 has a high degree of structural similarity to the substrate binding components of ABC transporters involved in the acquisition of methionine, and the protein was crystallized with l-methionine bound within the cleft of its two globular lobes (28). One of the best-studied bacterial methionine uptake systems is the high-affinity MetNIQ ABC transporter of Escherichia coli, in which the methionine binding component is encoded by *metQ* (29–32). The gonococcal homologue of GNA1946 is located in the outer membrane, was expressed by all 36 N. gonorrhoeae strains investigated, and is able to induce bactericidal antibodies (18), but it has not been characterized in detail. In this study, we investigated the function of the gonococcal GNA1946 homologue, which we propose be renamed MetQ, and assessed its potential as a gonococcal vaccine target.

## RESULTS

### Sequence analysis of the gonococcal *gna1946* and *metQ* homologue.

**FIG 1:**
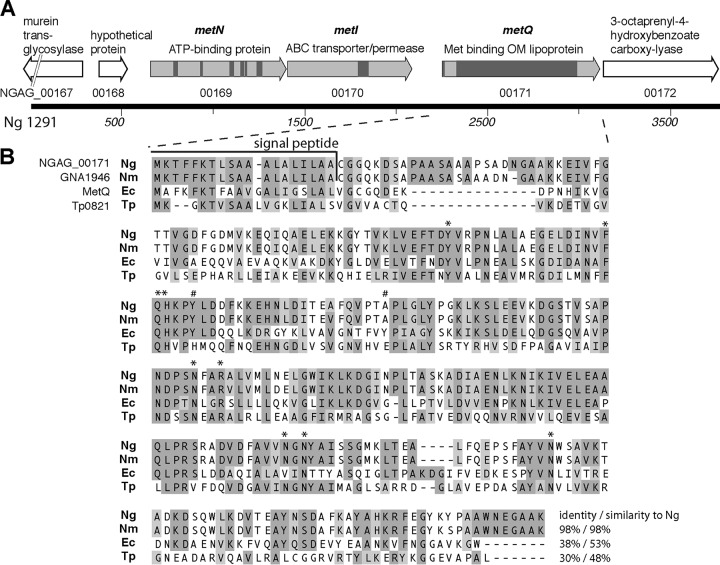
Schematic of the gonococcal locus encoding MetQ. (A) Structure of the NGAG_00167-NGAG_00172 locus from N. gonorrhoeae strain 1291 containing the *metNIQ* operon (light gray) and flanking genes (white). Arrows represent open reading frames, with the genome locus tag being shown below the arrow and the known/probable function being shown above. The locations of conserved motifs and domains in *metNIQ*, as described in the text, are shown by dark gray boxes. OM, outer membrane. (B) Alignment of the sequence of the putative MetQ (NGAG_00171 gene locus) of Neisseria gonorrhoeae (Ng) with the GNA1946 sequence of Neisseria meningitidis (Nm), the MetQ sequence of Escherichia coli (Ec), and the Tp32 sequence (Tp0821 gene locus) of Treponema pallidum (Tp). The percent identity and the percent similarity of each protein to the MetQ of N. gonorrhoeae are indicated. The signal peptides of N. gonorrhoeae and N. meningitidis are shown, as are the amino acids involved in binding to methionine in the T. pallidum structure (*, identical in N. gonorrhoeae; #, not conserved in N. gonorrhoeae).

 The NGAG_00171 gene of N. gonorrhoeae strain 1291 (Fig. 1A) (NGO2139 in N. gonorrhoeae FA1090) encodes a putative protein of 288 amino acids, which, on the basis of sequence and structural homology, is predicted to encode the methionine binding component of an ATP-binding cassette (ABC) transport system. The NGAG_00171 gene product has a high level of sequence identity to GNA1946 of N. meningitidis strain MC58 (98% identity) and to MetQ of Escherichia coli (38% identity, 53% similarity) (Fig. 1B), and we propose that it be named MetQ. Structural analysis of recombinant GNA1946 from N. meningitidis revealed binding to l-methionine (28), and MetQ of E. coli has been well characterized as a high-affinity methionine binding component of an ABC transport system encoded by the *metNIQ* operon (29–33). ABC transporters have a common basic architecture with an ATP-binding component containing two nucleotide-binding domains (NBDs) and a transporter/permease component containing two transmembrane domains (TMDs), and the substrate is typically delivered to this core importer complex via a substrate binding protein (SBP) (34). Similar to the E. coli *metNIQ* locus, the gonococcal *metQ* gene is located downstream of and appears to be part of an operon with two genes that are predicted to encode the ATP-binding protein (NGAG_00169, *metN*) and transmembrane transporter/permease (NGAG_00170, *metI*) of an ABC transport system (Fig. 1A). NGAG_00169 (*metN*) has 36% identity and 51% similarity to E. coli metN and contains all of the motifs typically found in NBDs that play a role in ATP binding, including the Walker A/P loop, Q loop/lid, ABC transporter signature motif, Walker B loop, D loop, and H loop/switch region. NGAG_00170 (*metI*) has 40% identity and 59% similarity to E. coli metI and contains the EAA motif (L loop) typical of TMDs (34) (Fig. 1A). NGAG_00171 (*metQ*) contains a domain that is conserved in the type 2 periplasmic binding fold (PBP2) superfamily of proteins (cl21456; amino acids [aa] 45 to 217; *P* = 1.18 × 10^−140^), which is characterized as a ligand-binding domain found in solute binding proteins, and the lipoprotein_9 protein family (Pfam03180; aa 46 to 281; *P* = 8.24 × 10^−104^), which is a family of bacterial lipoproteins that contains several antigenic members that may be involved in bacterial virulence. NGAG_00171 also contains a predicted lipoprotein signal peptide (aa 1 to 19) that contains a cysteine residue (Fig. 1B), which suggests localization in the outer membrane.

Analysis of the structure of the meningococcal GNA1946 protein (PDB accession number 3GXA; 98% identity to NGAG_00171) revealed a very high degree of structural similarity to the E. coli MetQ (PDB accession number 4YAH; 237 residues aligned between the structures with a root mean square deviation [RMSD] of 1.74 Å) and the Tp32 l-methionine binding protein of Treponema pallidum (PDB accession number 1XS5; 232 residues aligned between the structures with an RMSD of 1.65 Å). Eleven residues were identified as being involved in methionine binding by Tp32 (35), and nine of these are conserved in the gonococcal MetQ (Fig. 1B).

The *metQ* gene is highly conserved in the pathogenic Neisseria spp., with >99% nucleotide sequence identity being found among all publicly available sequences of N. gonorrhoeae (*n* = 20; Broad Institute and NCBI databases) and >95% nucleotide sequence identity being found among the sequences of available N. meningitidis strains (*n* = 1,381; Meningococcal Research Foundation and NCBI databases).

### Generation of MetQ knockout and complemented strains.

**FIG 2:**
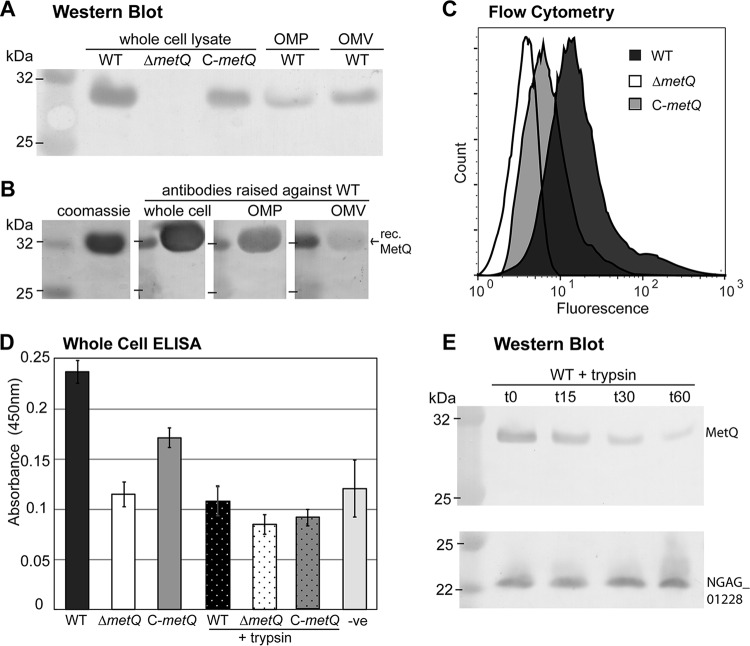
Cell surface localization of MetQ. (A) Western blot of the N. gonorrhoeae 1291 wild type (WT), *metQ* knockout (Δ*metQ*), and complemented (C-*metQ*) strains using polyclonal anti-MetQ antibodies. The samples analyzed included whole-cell lysates, OMP fractions, and OMVs. (B) Coomassie-stained SDS-polyacrylamide gel and Western blot of recombinant His-tagged MetQ (rec. MetQ) probed with polyclonal antibodies raised against either heat-inactivated whole cells, OMPs, or OMVs of the N. gonorrhoeae 1291 wild type. (C) Flow cytometry of whole cells of the N. gonorrhoeae wild type, Δ*metQ*, and C-*metQ* strains, with the expression of MetQ on the cell surface being determined by the detection of binding of polyclonal anti-MetQ antibody and secondary Alexa Fluor 488 anti-mouse immunoglobulin antibody. (D) Whole-cell ELISAs of untreated and trypsin-treated (+trypsin) N. gonorrhoeae wild type, Δ*metQ*, and C-*metQ* strains using polyclonal anti-MetQ antibodies. The results for the negative control (−ve), containing secondary antibody only, are also shown. The graph shows the average absorbance at 450 nm from three independent replicates ±1 standard deviation. By Student's *t* test, *P* was <0.00001 for the Δ*metQ* strain, the wild-type strain treated with trypsin, or the C-*metQ* strain treated with trypsin versus the wild type; *P* was <0.002 for the Δ*metQ* strain versus the wild type or C-*metQ* strain; and *P* was >0.4 for the Δ*metQ* strain, the wild-type strain treated with trypsin, or the C-*metQ* strain treated with trypsin versus the negative control. (E) Western blot analysis of whole-cell lysates of the N. gonorrhoeae wild type treated with trypsin for 0, 15, 30, or 60 min and probed with antibodies to MetQ or the periplasmic protein NGAG_01228 (meningococcal GNA1030/NUbp homologue).

To characterize the function of the gonococcal MetQ homologue, an isogenic mutant of N. gonorrhoeae strain 1291 (N. gonorrhoeae Δ*metQ*) was generated by insertion of a kanamycin resistance cassette into the open reading frame of the NGAG_00171 gene, and a complemented strain (N. gonorrhoeae C-*metQ*) was generated by reintroducing a single copy of the NGAG_00171 gene into the genome in *trans*. A recombinant His-tagged MetQ protein was generated in E. coli, and polyclonal antibodies against MetQ were raised in mice. Western blot analysis of whole-cell lysates confirmed the expression of MetQ in the wild-type strain, with a single band being detected by the anti-MetQ antibodies. The complemented strain had slightly lower levels of MetQ expression than the wild-type strain, and no MetQ expression was detected in the mutant strain (Fig. 2A and S1 in the supplemental material). MetQ expression was further investigated by quantitative real-time PCR (qRT-PCR) analysis using RNA prepared from the wild-type, knockout, and complemented strains. No amplicon was detected in the Δ*metQ* knockout strain, suggesting the complete absence of transcription, while complementation restored the expression of *metQ* to a level similar to that in the wild-type strain. qRT-PCR also showed that the levels of expression of the upstream (NGAG_00170) and downstream (NGAG_00172) genes were not significantly altered in the mutant strain compared to the wild type (data not shown). The mutant strain had no growth defect relative to the wild-type and complemented strains in GC-IsoVitaleX, brain heart infusion-IsoVitaleX, or RPMI-IsoVitaleX (with or without methionine) broth (data not shown).

### Gonococcal MetQ is localized on the surface of N. gonorrhoeae.

The majority of solute binding proteins of ABC transporters are located in the periplasm of Gram-negative bacteria (34); however, several examples of surface-localized solute binding proteins have been characterized (20, 23–25). MetQ has been identified, via proteomic analysis, in outer membrane protein (OMP) and outer membrane vesicle (OMV) preparations of N. gonorrhoeae (18, 36) and N. meningitidis (37). In order to investigate the surface exposure of MetQ on N. gonorrhoeae, several approaches were used. The localization of MetQ to the outer membrane was confirmed by Western blotting of OMP and OMV preparations of the wild-type strain with antibodies raised against recombinant MetQ (Fig. 2A). Immunization of mice with whole bacteria or OMP or OMV preparations also generated antibodies that recognized recombinant MetQ by Western blotting (Fig. 2B). The weaker signal seen with antibodies raised against OMVs was possibly due to MetQ being less abundant and/or less immunogenic when presented in OMVs. Flow cytometry (Fig. 2C), whole-cell enzyme-linked immunosorbent assays (ELISAs) (Fig. 2D) and fluorescence microscopy (data not shown) demonstrated that MetQ is surface exposed, with MetQ being detected on the surface of the wild-type and complemented strains but not the Δ*metQ* mutant strain. In addition, whole-cell ELISAs of gonococcal strains treated with trypsin showed a significant reduction of antibody binding to the wild-type and complemented strains (*P* = 0.013 and < 0.0001, respectively) to a level that was comparable to the level of binding to the untreated Δ*metQ* strain (Fig. 2D). Furthermore, Western blot analysis of trypsinized whole wild-type bacteria demonstrated a gradual reduction of the MetQ band intensity over 60 min of treatment, while no change was seen for the periplasmic protein NGAG_01228 (meningococcal GNA1030/NUpb homologue) (Fig. 2E). Treatment of bacteria with trypsin did not affect cell viability, as there was no significant difference in CFU counts before and after trypsin treatment (2.2 × 10^9^ CFU at time zero versus 1.9 × 10^9^ CFU at 60 min; *P* = 0.4). Some MetQ was still detected after trypsin treatment, indicating that a portion of MetQ may be localized inside the membrane, as suggested by Zielke et al. (18). Together, these data confirm the localization of MetQ on the surface of gonococcal cells.

### Gonococcal MetQ binds l-methionine.

**FIG 3:**
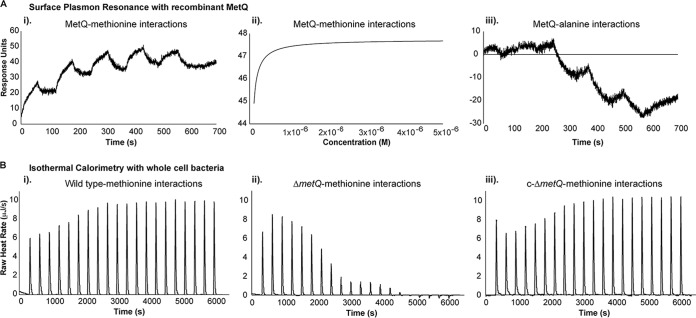
l-Methionine binding to MetQ. (A) (i and ii) SPR sensograms (i) and line-of-best-fit curve (ii) of l-methionine binding to immobilized recombinant MetQ protein. The line of best fit was generated in Biacore T100 Evaluation software, with χ^2^ values for all experiments falling under 0.1 of the maximum response units (*R*_max_). Single-cycle kinetics were used to generate the *K_D_* of the interactions between MetQ and the l-methionine substrate. (iii) SPR sensograms showing no interaction between the negative control (alanine) and immobilized MetQ. (B) ITC of the interaction of the N. gonorrhoeae 1291 wild-type (i), *metQ* knockout (Δ*metQ*) (ii), and complemented (C-*metQ*) (iii) strains with l-methionine. The graphs show heat changes upon injection of l-methionine, which is dependent on the presence of MetQ.

The gonococcal MetQ is predicted to encode the solute binding component of a methionine ABC transport, on the basis of sequence homology to other methionine transporters. To investigate the specificity and the kinetics of ligand binding by MetQ, surface plasmon resonance (SPR) and isothermal titration calorimetry (ITC) analyses were performed. For SPR analysis, recombinant MetQ was immobilized on the sensor chip and free amino acids were flowed over the immobilized protein. MetQ bound to l-methionine with a high affinity, with the *K_D_* (equilibrium dissociation constant) being 7.48 ± 0.26 nM, while no detectable binding to the negative control, alanine, was observed (Fig. 3A). For ITC, calorimetric titration of whole cells of the wild-type, Δ*metQ*, and C-*metQ* strains of bacteria with 0.3 mM l-methionine was performed. Comparison of the findings for the wild-type and complemented strains to those for the Δ*metQ* strain indicated the presence of two distinct interactions with l-methionine by the wild-type and complemented strains, whereas only one interaction by the Δ*metQ* strain was seen (Fig. 3B). This suggests the presence of a second methionine binding protein in N. gonorrhoeae, in addition to MetQ, which is similar to the findings for E. coli, where two methionine transporters have been reported (30). These data confirm the role of MetQ as a specific and high-affinity methionine binding protein.

### MetQ is involved in adherence to cervical epithelial cells.

**FIG 4:**
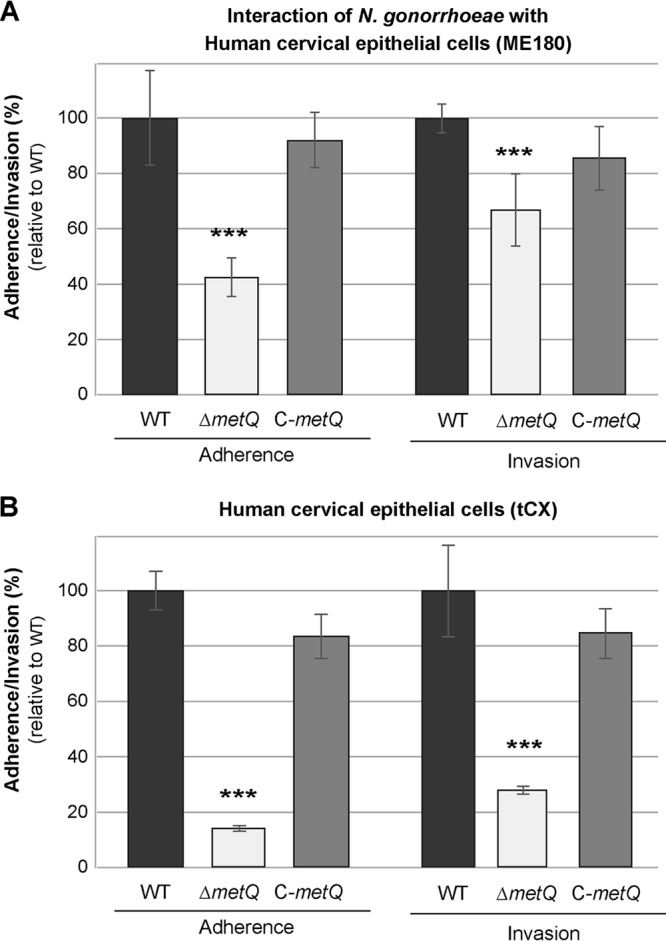
Role of MetQ in adherence to cervical epithelial cells. Adherence and invasion of ME180 (A) and tCX (B) human cervical epithelial cells by the N. gonorrhoeae 1291 wild-type (WT), *metQ* knockout (Δ*metQ*), and complemented (C-*metQ*) strains. Data represent the average percent adherence or invasion for triplicate samples as a percentage of that for the inoculum and are shown relative to the results obtained with the wild-type strain (the results for the wild type, set at 100%, are 2.9 × 10^5^ adherent CFU and 1.1 × 10^4^ invasive CFU for ME180 cells and 5.5 × 10^6^ adherent CFU and 1.3 × 10^5^ invasive CFU for tCX cells). Error bars represent ±1 standard deviation. ***, *P* ≤ 0.001 for the Δ*metQ* strain relative to the wild type, using a two-tailed Student's *t* test. Experiments were performed on at least three occasions, and representative results are shown.

The substrate binding proteins of several ABC transporters have been characterized as adhesins (21, 25). In order to determine whether the gonococcal MetQ plays a role in the colonization of host cells, assays of adherence to and invasion of cervical epithelial cells (transformed primary tCX cells and cancer ME180 cells) were performed with the wild-type, Δ*metQ* mutant, and C-*metQ* complemented strains. The Δ*metQ* strain had a 2.4-fold lower level of adherence to ME180 cells and a 1.5-fold lower level of invasion of ME180 cells than the wild-type strain (Fig. 4A) (*P* < 0.001). Similarly, the Δ*metQ* strain had a 7.7-fold lower level of adherence to tCX cells and a 3.4-fold lower level of invasion of tCX cells than the wild-type strain (Fig. 4B; *P* < 0.001). Near wild-type levels of adherence and invasion were restored in the complemented strain (Fig. 4A and B).

### MetQ is involved in gonococcal survival in primary monocytes and macrophages and normal human serum.

**FIG 5:**
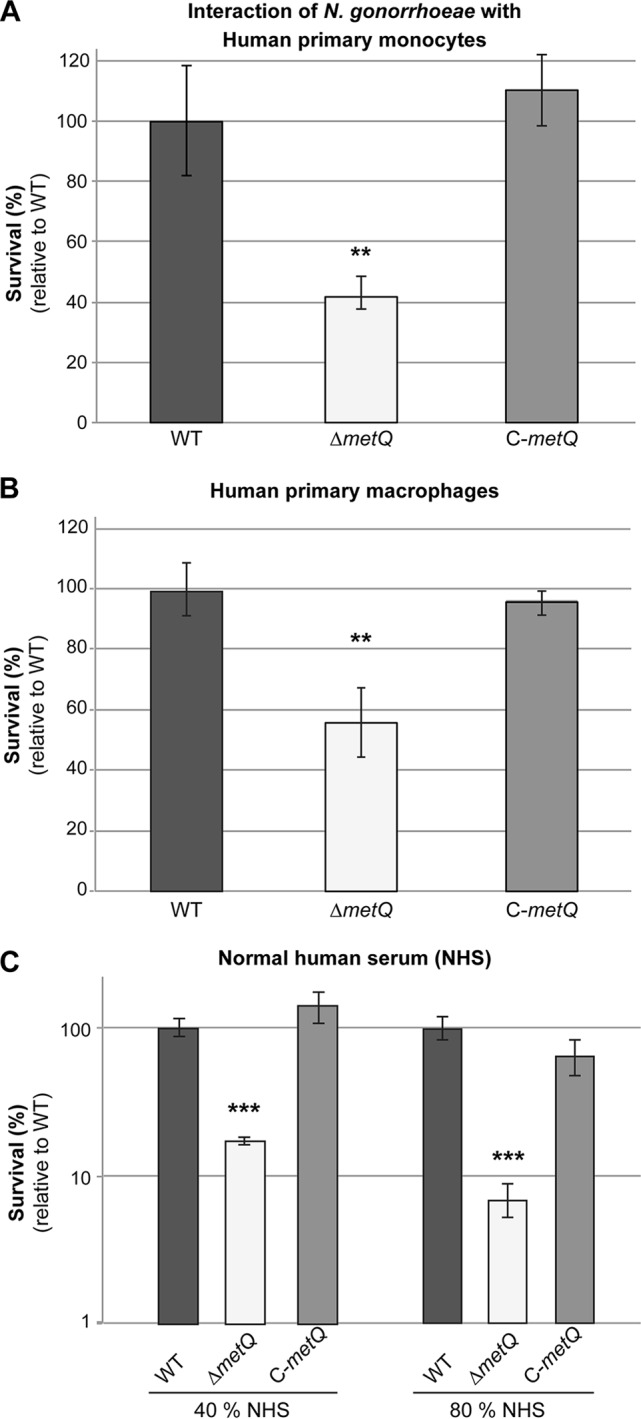
Role of MetQ in survival in monocytes, macrophages, and human serum. Survival of the N. gonorrhoeae 1291 wild-type (WT), *metQ* knockout (Δ*metQ*), and complemented (C-*metQ*) strains with primary monocytes (A), activated macrophages (B), and normal human serum (NHS) (C). Data represent the average percent survival for triplicate samples as a percentage of the inoculum size and are shown relative to the results obtained with the wild-type strain (the results for the wild type, set at 100%, are 2.4 × 10^4^ CFU for monocytes, 1.2 × 10^5^ CFU for macrophages, 7.0 × 10^4^ CFU for 40% normal human serum, and 1.9 × 10^4^ CFU for 80% normal human serum). Error bars represent ±1 standard deviation. **, *P* ≤ 0.01 for the Δ*metQ* strain relative to the wild type, using a two-tailed Student's *t* test; ***, *P* ≤ 0.001 for the Δ*metQ* strain relative to the wild type, using a two-tailed Student's *t* test. Experiments were performed on at least three occasions, and representative results are shown.

Assays of infection of primary human phagocytes (monocytes, activated macrophages, and neutrophils) and normal human serum were conducted with the wild-type, Δ*metQ* mutant, and C-*metQ* complemented strains to determine whether MetQ plays a role in protection from killing by immune cells or complement factors. The Δ*metQ* strain had a 2.3-fold reduced survival in primary monocytes (Fig. 5A) (*P* = 0.006) and a 1.5-fold reduced survival in activated macrophages (Fig. 5B) (*P* = 0.007) relative to the wild type, while near wild-type levels of survival were restored in the complemented strain. The Δ*metQ* strain also had significantly reduced rates of survival in normal human serum from two donors relative to those of the wild type and complemented strains, with a 5.7-fold lower rate of survival than the wild type in 40% serum and a 14.7-fold lower rate of survival than the wild type in 80% serum (Fig. 5C) (*P* < 0.001; data for donor 2 are not shown). No difference in survival between the three strains incubated in broth only or in heat-inactivated 80% serum was seen (data not shown), indicating that active complement is involved in the reduced survival of the Δ*metQ* strain.

The wild-type, mutant, and complemented strains all showed similar levels of survival in primary human neutrophils and under conditions of detergent stress (Triton X-100) and oxidative stress (hydrogen peroxide), suggesting that the phenotypes described above for the Δ*metQ* strain are not due to a general altered membrane structure or altered fitness (data not shown).

### Anti-MetQ antibodies are bactericidal and are able to reduce adherence to cervical epithelial cells.

**FIG 6:**
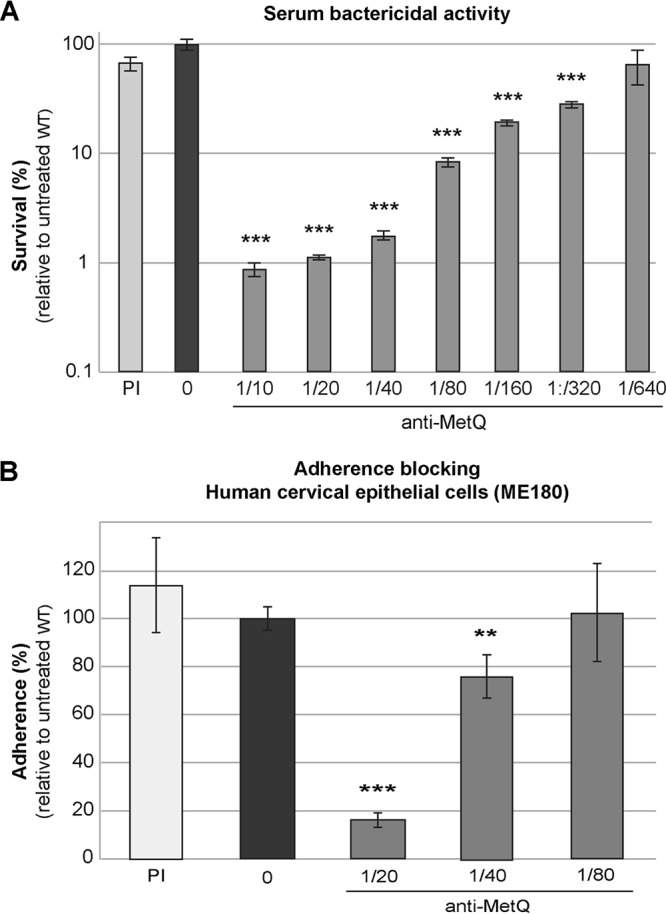
Functional activity of anti-MetQ antibodies. (A) Serum bactericidal activity. The survival of the wild-type (WT) strain in the presence of a 1/10 dilution of preimmune serum (PI) or 2-fold dilutions of heat-inactivated anti-MetQ mouse polyclonal serum with 10% normal human serum (preabsorbed with N. gonorrhoeae) as a source of complement is shown. (B) Blocking of adherence to cervical epithelial cells. The adherence of the wild-type strain in the presence of a 1/20 dilution of preimmune serum or 2-fold dilutions of heat-inactivated anti-MetQ mouse polyclonal sera is shown. Data represent the average survival (A) or adherence (B) for triplicate samples as a percentage of the inoculum size and the adherence of the inoculum and are shown relative to the result obtained with the untreated wild-type strain (the results for the untreated wild type, set at 100%, are 1.8 × 10^5^ CFU for serum bactericidal activity and 9.1 × 10^4^ CFU for adherence). Error bars represent ±1 standard deviation. In both panels A and B, there was a statistically significant difference between groups, as determined by one-way ANOVA [*F*(9, 20) = 46.58 and *P* = 1.8 × 10^−11^ and *F*(6, 21) = 32.5 and *P* = 1.4 × 10^−9^, respectively]. **, *P* ≤ 0.01 relative to the untreated wild type, using a two-tailed Student's *t* test; ***, *P* ≤ 0.001, relative to the untreated wild type, using a two-tailed Student's *t* test. Experiments were performed on at least three occasions, and representative results are shown.

The functional activity of the mouse anti-MetQ polyclonal antibodies was investigated in terms of their ability to mediate serum bactericidal activity (SBA) and block the adherence of N. gonorrhoeae to cervical epithelial cells. The specificity of the mouse serum for MetQ was shown by Western blotting, with only a single band being detected by the anti-MetQ polyclonal antibodies (Fig. 2A and S1). The complement-mediated antibody-dependent serum bactericidal activity of the anti-MetQ antibodies was tested against the N. gonorrhoeae 1291 wild type using 10% human serum (preabsorbed with N. gonorrhoeae) as the complement source. Antibody concentration-dependent killing was seen with a bactericidal titer of 320 (the reciprocal of the final serum dilution giving ≥50% killing at 30 min) (Fig. 6A). In addition, preincubation of N. gonorrhoeae 1291 with anti-MetQ antibodies resulted in a concentration-dependent decease in adherence to ME180 cervical epithelial cells relative to that for the no-serum or preimmune serum controls, with 6.1- and 1.5-fold decreased adherence relative to that for the no-serum control being seen at 1/20 and 1/40 dilutions of anti-MetQ antibodies, respectively (Fig. 6B) (*P* < 0.01).

## DISCUSSION

N. gonorrhoeae is considered an urgent threat to public health due to its increasing incidence and widespread antibiotic resistance (3, 38). As such, the development of alternative treatment options and a vaccine for N. gonorrhoeae has become a priority. Bacterial ABC transporters constitute attractive targets for vaccines and drug development due to their central role in nutrient acquisition (39), and the solute binding protein components of several ABC transporters have been described to be effective vaccine targets in animal models, including the PstS phosphate transport protein of Mycobacterium tuberculosis (40), as well as the PsaA manganese transporter (23), the PiuA and PiaA iron transporters (41), and the PotD polyamine transporter (42) of S. pneumoniae. In this study, we have characterized the functional role of the methionine binding protein of an ABC transporter (encoded by NGA_00171, which we have called *metQ*) of N. gonorrhoeae and highlighted its potential as a gonococcal vaccine candidate. We have shown that the gonococcal MetQ is highly conserved in all strains investigated to date, it is localized on the bacterial surface, it binds l-methionine with a high affinity, and it is involved in gonococcal adherence to epithelial cells and survival in human monocytes, macrophages, and serum. Furthermore, we have shown that antibodies raised against MetQ are bactericidal and are able to reduce adherence to cervical epithelial cells.

Nutrient acquisition mechanisms, such as ABC transporters, are often linked to bacterial pathogenesis (19), since the size and scarcity of certain nutrients require solute-specific, high-affinity outer membrane receptors and transport systems to move the compounds into the periplasmic space. Methionine is one of the least abundant amino acids in physiological fluids (∼33.4 μM l-methionine in human serum [43] and ∼23 μM in human uterine fluid [44]), but it is essential for several cellular functions. It is the universal N-terminal amino acid of proteins and a key component of *S*-adenosylmethionine (the main cellular carrier of methyl groups, which is required for the biosynthesis of phospholipids and nucleic acids) and can act as an endogenous antioxidant due to its ability to undergo reversible oxidation and reduction (45, 46). In keeping with these important roles, methionine uptake and synthesis are involved in virulence in bacterial pathogens, such as Salmonella enterica (47), Haemophilus influenzae (48), and Streptococcus pneumoniae (49). Methionine is also synthesized *de novo* from aspartate by most microorganisms, which involves a series of reactions, including inorganic sulfate assimilation and synthesis of cysteine or homocysteine (46). N. gonorrhoeae has an absolute requirement for cysteine or cystine (50), and while methionine auxotrophy was very common in the sulfonamide era in sulfonamide-resistant bacteria, it became rare in the penicillin era and had largely disappeared by 1970 (51, 52).

ABC transporters are the primary methionine uptake systems in several bacteria and include MetNIQ (MetD) of E. coli (29–32) and MetQNP of S. pneumoniae (49). The NGAG_00169-NGAG_00171 locus in N. gonorrhoeae strain 1291 is predicted to encode a similar methionine uptake system, which we propose be renamed MetNIQ. Sequence and structural analyses revealed a high level of similarity between the gonococcal MetQ and the E. coli MetQ, and using biointeraction analyses (SPR and ITC), we confirmed that MetQ is a high-affinity l-methionine receptor. The Δ*metQ* strain did not have a growth defect in medium lacking l-methionine, which suggests that adequate methionine is available from endogenous synthesis to support *in vitro* growth and/or, as indicated by ITC analysis, that N. gonorrhoeae has an additional l-methionine receptor. A similar situation is seen in E. coli, which contains the high-affinity MetNIQ ABC transporter as well as the lower-affinity MetP system (29–32). E. coli MetNIQ mutants have growth and internal methionine concentrations similar to those of the wild type (30).

ABC transporters consist of a transmembrane permease and ATP-binding components that are located in the inner membrane and cytoplasm, respectively, and that are associated with a high-affinity solute binding protein that is typically located in the periplasm of Gram-negative bacteria or the cell surface of Gram-positive bacteria. However, several surface-localized binding proteins that are associated with ABC transporters in bacteria, including Mycobacterium tuberculosis (24) and Campylobacter jejuni (53), have been characterized. MetQ has a lipoprotein signal peptide that suggests outer membrane localization, and the results of our immunoblotting, ELISA, flow cytometry, and microscopy analyses confirm that the gonococcal MetQ is also located on the cell surface. This is consistent with the results of a recent proteomics analysis that indicated that MetQ is found predominantly in the cell envelopes and is also present in outer membrane vesicles and the cytosol but not in the soluble fraction of the supernatant (18). The surface localization of the MetQ lipoprotein supports its potential as a vaccine antigen that would be accessible to vaccine-induced antibodies but raises questions as to the mechanism by which it relays methionine to its cognate ABC transporter components in the inner membrane. Most bacterial lipoproteins are located on the periplasmic side of the outer membrane, with others being located on the inner membrane. However, a growing number of surface-exposed lipoproteins have been described (54). Furthermore, Pal of E. coli (55) and the Pal homologue, P6, of nontypeable Haemophilus influenzae (56) are lipoproteins with dual orientations in the outer membrane, existing as a surface-exposed protein and a periplasmic protein. Our findings and recent data from Zielke et al. (18) indicate a possible dual localization of MetQ, which supports its proposed dual functions in methionine uptake and adherence to host epithelial cells.

During colonization, N. gonorrhoeae adheres to and invades mucosal epithelial cells, and acute gonorrhea is characterized by an inflammatory exudate that contains numerous polymorphonuclear leukocytes (PMNs), as well as macrophages and exfoliated epithelial cells (7). The inflammatory response is also believed to result in the presence of complement factors on mucosal surfaces (57). N. gonorrhoeae has numerous mechanisms to evade these host defenses and survive in epithelial cells (58–60), neutrophils (61), macrophages (62), and serum (63). Using *in vitro* and *ex vivo* assays to model different aspects of infection, we have shown that MetQ plays a role in various aspects of gonococcal colonization and disease. The Δ*metQ* knockout strain was impaired in its ability to adhere to and invade human cervical epithelial cells compared to the wild type. Previous studies have shown that surface-exposed lipoprotein components of ABC-type transporters, for example, the manganese binding protein PsaA of Streptococcus pneumoniae, which is a potential vaccine component (21, 64), and CD0873 of Clostridium difficile (a putative sugar-family solute binding protein) (25), are often involved in the adherence of pathogenic bacteria to host cells. In addition, the Δ*metQ* mutant strain also had reduced survival in primary monocytes and activated macrophages but not neutrophils. In untreated infections, polymorphonuclear leukocytes are gradually replaced by mononuclear cells (7), suggesting that MetQ may play a more important role during chronic infection. The Δ*metQ* strain was also more susceptible to killing by normal human serum at various concentrations than the wild type. Complement is an important part of the mucosal defense system that would be encountered by N. gonorrhoeae during natural infection (57, 65). The concentrations of complement factors at mucosal surfaces are predicted to be ∼10% of those found in serum (66), and it is expected that N. gonorrhoeae may encounter differing levels of serum bactericidal activity, depending on the stage of the infection and the level of inflammation (65).

We have shown that *metQ* is highly conserved in the gonococcal strains investigated, and previous work suggests that it is expressed during various stages of infection. Zielke et al. have shown that MetQ is constitutively expressed during 6 h of aerobic growth and is uniformly expressed during growth in normal medium compared to its expression during growth in human serum under conditions of iron depletion and oxygen deprivation (18). McClure et al. also showed that there is no difference in MetQ expression between bacteria grown in normal medium and bacteria from samples derived directly from cervicovaginal lavage specimens from infected women (67). On the basis of the findings of our bioinformatic analysis, MetQ is not predicted to have phase-variable expression (i.e., random, reversible on-off, or graded switching of expression), as no repetitive DNA tracts were identified in the putative promoter region or open reading frame of the gene, and it has not been identified in the N. gonorrhoeae or N. meningitidis phasevarions studied to date (68, 69). Interestingly, Echenique-Rivera et al. found that expression of the meningococcal MetQ homologue NMB1946/GNA1946 was upregulated in bacteria incubated in human blood (70), and a knockout mutant of the MetQ homologue of S. pneumoniae was found to have a growth defect in blood (49) and to be less competitive than the wild-type strain in mouse infection models of pneumonia and sepsis (71). The direct function and mechanism of action of MetQ during interactions with and survival in epithelial cells, mononuclear phagocytes, and human serum are unknown, but it may have a role in methionine binding and uptake for the nutrient and/or antioxidant function or an as yet uncharacterized function.

Given the increasing need for a gonococcal vaccine, the identification and characterization of novel vaccine targets are essential. To this end, we have shown that MetQ is immunogenic in mice and anti-MetQ antibodies are bactericidal against N. gonorrhoeae strain 1291 with a bactericidal titer of 320. This is in keeping with recent results from Zielke et al. that rabbit antibodies raised against MetQ from N. gonorrhoeae strain FA1090 have bactericidal titers of 20 for the homologous N. gonorrhoeae strain FA1090 and 400 for the heterologous strain MS11 (18). Furthermore, we have shown that anti-MetQ antibodies are able to reduce adherence to cervical epithelial cells, which may translate into the ability of vaccine-induced antibodies to reduce the level of colonization by N. gonorrhoeae. Preclinical development and the evaluation of gonococcal vaccine antigens have been hampered by the fact that there are no known correlates of protection for N. gonorrhoeae and there is no animal model that accurately mimics the complexity of human infection and disease (reviewed in reference 2). However, a vaccine consisting of a combination of the most promising antigens, based on data from *in vitro* model systems of infection, could have a significant impact on the levels of infection, transmission, and/or disease and should be advanced to clinical trials as soon as possible.

In conclusion, our findings indicate that MetQ is an attractive vaccine candidate due to its ability to induce antibodies that are bactericidal and able to block adherence to host cells, which are characteristics that may allow it to play an important role in reducing gonococcal transmission and disease.

## MATERIALS AND METHODS

### Bacterial strains and growth conditions.

N. gonorrhoeae strain 1291 (a male gonococcal urethritis isolate, provided courtesy of M. A. Apicella) was grown at 37°C with 5% CO_2_ on GC agar plates (Oxoid) supplemented with IsoVitaleX (Becton Dickinson) for ∼16 h. Escherichia coli strains DH5α and BL21 were grown in Luria-Bertani broth or agar at 37°C. Kanamycin, ampicillin, and spectinomycin were used as required at final concentration of 50 μg/ml, 100 μg/ml, and 100 μg/ml, respectively. Growth rate experiments were performed in GC broth supplemented with IsoVitaleX, as described previously (72).

### Sequence analysis.

The distribution of *metQ* in N. gonorrhoeae genomes (available at the Neisseria gonorrhoeae Comparative Initiative, Broad Institute [broadinstitute.org], and the NCBI Genome Database [http://www.ncbi.nlm.nih.gov/genome/864]) was investigated using BLASTn and BLASTx searches with NGAG_00171 from the genome of N. gonorrhoeae 1291 (GenBank accession number NZ_DS999919), *gna1946* of N. meningitidis strain MC58 (GenBank accession number AF226499), and *metQ* of E. coli strain MG1655 (GenBank accession number NC_000913; locus tag, b0197). The structural similarity between the MetQ homologues was investigated using the SPalign server (73).

### Construction of N. gonorrhoeae 1291 *metQ* knockout and complemented strains.

The *metQ* knockout construct was made by cloning the NGAG_00171 gene from the genome of N. gonorrhoeae 1291 (using primers 5′-GCCGTCTGAACGCCGAATCCGGACGGC-3′ and 5′-GTATCGCACACACGCTGTCGCTGTTCGG-3′) into the pGEM-T Easy vector (Promega). A kanamycin resistance cassette (pUC4Kan; Amersham Biosciences) was then inserted into a unique BamHI restriction site introduced into the middle of the *metQ* open reading frame (via inverse PCR with primers 5′-GGATCCGCCCAACGACCCGTCCAACTTCG-3′ and 5′-GGATCCGCGGATACGGTGCTGCCGTC-3′; restriction enzyme sites added for cloning purposes are underlined here and below). The complementation construct was made by cloning *metQ* from the genome of N. gonorrhoeae 1291 (using primers 5′-CTTAAGCCGTCTGAACGCCGAATCCG-3′ and 5′-CCCGGGGCCTTATTTGGCTGCGCCTTC-3′) into the pCTS32 complementation plasmid (74) between the AflII and SmaI restriction sites. The knockout and complementation constructs were linearized with NotI and NcoI, respectively, and transformed into naturally competent N. gonorrhoeae 1291 (the wild-type and mutant strains, respectively) using standard protocols (75). Transformants were confirmed using PCR and Western blot analysis. The wild-type, mutant (Δ*metQ*), and complemented (C-*metQ*) strains expressed similar Opa, pilus, and lipopolysaccharide (LOS) profiles, as determined by phase-contrast microscopy analysis of the colony morphology (all strains were Opa and pilus positive) and SDS-PAGE (by which the strains had LOS of the same molecular weight and abundance) (data not shown).

### qRT-PCR analysis.

RNA was extracted from the N. gonorrhoeae wild-type, mutant, and complemented strains using an RNeasy minikit (Qiagen), and cDNA was generated with Moloney murine leukemia virus reverse transcriptase (New England BioLabs). Quantitative real-time PCR (qRT-PCR) was performed using a SYBR green JumpStart kit (Sigma-Aldrich). The relative expression of the gene *metQ* (primers 5′-AAAGACGGCAGCACCGTATC-3′ and 5′-CGATGTCGGCTTTGGATGC-3′), the downstream gene NGAG_00172 (primers 5′-GCTTCGGCGACAATCTCTTG-3′ and 5′-CCAAATGGGCAAGGTTCAGC-3′), and the upstream gene NGAG_00170 (primers 5′-GGCGTGTTGCTCTTCGTAAC-3′ and 5′-TGAGGTTGACGAGGTTGTCG-3′) was determined using the ΔΔ*C_T_* threshold cycle (*C_T_*) quantification method with 16S rRNA as a reference.

### Recombinant protein expression.

E. coli BL21(DE3) was transformed with the pET21b plasmid carrying the full-length *gna1946* sequence from N. meningitidis (26) (provided courtesy of M. Pizza). The expression strain was then cultured in LB broth supplemented with ampicillin to an optical density at 600 nm (OD_600_) of 0.4 at 25°C. Isopropyl-1-thiogalactopyranoside was added to a final concentration of 100 mM, and the culture was further incubated at 25°C overnight. Bacterial cells were pelleted and resuspended in phosphate buffer (50 mM sodium phosphate, 300 mM sodium chloride, pH 7) containing lysozyme (0.1 mg/ml). Bacterial cells were lysed by sonication, and soluble fractions were separated via centrifugation. The collected fraction was then applied to an equilibrated Talon resin (Clontech) and incubated at 4°C overnight. The resin was washed three times with phosphate buffer containing 20 mM imidazole before His-tagged MetQ was eluted with 150 mM imidazole.

### OMP preparation.

N. gonorrhoeae was harvested from a plate in which it had been cultured overnight into 10 mM Tris-HCl (pH 7.4) containing lysozyme (1 mg/ml) and incubated on ice for 10 min, sonicated, and then centrifuged (5,000 × *g*, 10 min, 4°C). Sodium lauroyl sarcosinate (final concentration, 1% [wt/vol]) was added to the soluble fraction, and the mixture was incubated at room temperature for 20 min and then centrifuged (100,000 × *g*, 1 h, 4°C). The pellet was resuspended in 1% sodium lauroyl sarcosinate, incubated at room temperature for 20 min, and centrifuged (100,000 × *g*, 1 h, 4°C). The pellet containing the gonococcal outer membrane proteins (OMPs) was resuspended in phosphate-buffered saline (PBS) containing 0.1% SDS. The protein concentration was measured using a bicinchoninic acid (BCA) protein assay (Thermo Scientific).

### OMV preparation.

Naturally secreted N. gonorrhoeae outer membrane vesicles (OMVs) were harvested from a 6-h culture (GC broth; OD_600_, ∼0.8) by brief centrifugation (5,000 × *g*) and subsequent filtration of the supernatant (filter pore size, 0.22 μm). The filtrate was centrifuged (100,000 × *g*, 1 h, 4°C), and the pellet containing OMV was washed three times with PBS. The pellet was solubilized in PBS containing 0.2% SDS. The protein concentration was measured using a BCA protein assay (Thermo Scientific).

### Generation of polyclonal antibodies.

Groups of five 3-week-old BALB/c female mice (Animal Resources Centre, WA, Australia) were immunized subcutaneously with either 50 μg of recombinant MetQ protein or 20 μg of OMP, OMV, or fixed whole-cell N. gonorrhoeae 1291 at day 1 (in 0.1 ml of Freund's complete adjuvant; Sigma-Aldrich) and days 7, 14, 21, and 35 (in Freund's incomplete adjuvant; Sigma-Aldrich), in accordance with Griffith University Animal Ethics Committee and institutional guidelines. Animal blood was collected on day 42, and serum was collected via centrifugation. Serum titers were determined using an ELISA.

### Analysis of MetQ expression.

Western blot analysis of MetQ expression in whole-cell lysates of N. gonorrhoeae was performed using standard methods. Briefly, cell lysates were prepared after overnight growth on agar plates, 10 μl (equivalent to a final OD_600_ of 5, or ∼5 × 10^6^ CFU) was loaded on a 4 to 12% bis-Tris NuPAGE polyacrylamide gel (Life Technologies), and a primary antibody (anti-MetQ, anti-whole cell, anti-OMP, or anti-OMV [1:1,000]) and horseradish peroxidase (HRP)-conjugated anti-mouse immunoglobulin secondary antibody (Sigma-Aldrich) were used for protein detection. Duplicate gels were run and Coomassie stained to confirm equal loading of samples (data not shown). Antibodies to GNA1030/NUpb (NGAG_01228; provided courtesy of M. Pizza) (76, 77) were used as a periplasmic protein control.

ELISAs were performed with 96-well MaxiSorp (Nunc) plates coated with N. gonorrhoeae (∼1 × 10^7^ CFU). The plates were washed with PBS and blocked with PBS–1% bovine serum albumin (BSA) for 1 h. The plates were then incubated with anti-MetQ antibody (1:1,000, 1 h at room temperature), washed three times with PBS, incubated with secondary antibody (1:20,000 HRP-conjugated anti-mouse, 1 h), washed, and then developed with tetramethylbenzidine solution (Life Technologies). The reaction was stopped by adding 1 volume of 1 M HCl. Absorbance readings were taken at 450 nm using a Victor^3^ plate reader (PerkinElmer).

Trypsin digestion of surface-exposed proteins was achieved by treating bacteria (∼1 × 10^9^ CFU/ml in PBS) with 2.5% trypsin (Life Technologies) at 37°C for 30 min for ELISA or 15 to 60 min for Western blot analysis. The cells were then washed 3 times in PBS and processed as outlined above. Bacterial viability was determined by comparison of the CFU counts before and after trypsin treatment.

Flow cytometry analysis was performed using 2.5% formaldehyde-fixed N. gonorrhoeae strains (∼1 × 10^8^ CFU/ml). The bacteria were centrifuged, washed three times, and resuspended in PBS–1% BSA–0.01% Tween 20. The bacteria were incubated with anti-MetQ antibody (1:1,000, 30 min at room temperature), washed three times with PBS, then incubated with secondary antibody (1:1,000; Alexa Fluor 488-conjugated anti-mouse immunoglobulin, 30 min at room temperature), and, finally, washed three times with PBS. Analysis was carried out using a CyAn ADP analyzer and Kaluza analysis software (Beckman Coulter).

### SPR.

The affinity and kinetics of MetQ interactions with amino acids were investigated using a series S CM5 sensor chip on a Biacore T100 surface plasmon resonance (SPR) system (General Electric [GE]). The optimum condition for the immobilization of MetQ onto the CM5 chip was determined by a series of pH scouting experiments to be pH 4.0. MetQ (50 μg/ml in 10 mM sodium acetate, pH 4.0) was captured on flow cells 2, 3, and 4. No protein was immobilized on flow cell 1 (the chip surface neutralized with ethanolamine), which was used as a reference for nonspecific interactions of the ligand with the chip. Single-cycle kinetics were used to generate the *K_D_* of the interactions between MetQ and the l-methionine substrate (concentration range, 1 nM to 10 μM). The analyte (l-methionine) was run in degassed HBS-EP^+^ running buffer (GE) at a flow rate of 30 μl/min for a contact time of 60 s. A 10-min dissociation step was performed at the end of each cycle. Data analysis was performed using Biacore Evaluation software (GE).

### ITC.

Isothermal calorimetry (ITC) was used to investigate the interactions between N. gonorrhoeae and l-methionine. N. gonorrhoeae strains (170 μl of ∼1 × 10^10^ CFU/ml in PBS containing 0.01% sodium azide to inhibit metabolic functions) were loaded into the cell of a nano-ITC system (TA Instruments) and titrated against 0.3 mM l-methionine (25 2.5-μl injections at 300-s intervals) at 36°C, and the heat obtained after injection of l-methionine into the experiment buffer (PBS, 0.01% sodium azide) was subtracted from all data sets. To observe the kinetics of l-methionine interactions with N. gonorrhoeae, the calorimetric titration data for the Δ*metQ* strain were subtracted from those for the wild-type and complemented strains. The data were analyzed using the NanoAnalyze software package (TA Instruments). The *K_D_* of these interactions could not be determined due to the residual heat in the wild-type strain that was absent in the Δ*metQ* strain (i.e., the interaction did not approach zero after subtraction of the heat from the control injections).

### Phagocyte killing assays.

Primary monocytes and polymorphonuclear neutrophils (PMNs) were isolated from donor blood using Histopaque density medium (Sigma-Aldrich), as previously described (78). Cells were collected into minimal essential medium (MEM) alpha (Gibco) and seeded at 10^5^ cells per well in a tissue culture coated 24-well plate (Corning). Monocyte differentiation into activated macrophages was achieved by incubation with Salmonella enterica rough lipopolysaccharide (10 ng/ml, 16 h at 37°C; Sigma-Aldrich), followed by three washes with PBS. N. gonorrhoeae strains (∼1 × 10^5^ CFU in MEM alpha) were added to PMNs, monocytes, or activated macrophages at a multiplicity of infection (MOI) of 1. The plate was centrifuged (100 × *g*, 5 min) and incubated at 37°C for 5 min. The cells were gently washed in PBS to remove nonphagocytosed bacteria and incubated in fresh medium at 37°C for 1 h. The cells were then lysed with 0.5% saponin, scraped from the well, serially diluted, and plated to determine the number of bacterial CFU. For this assay and all assays described below, experiments were performed in triplicate on at least three occasions, and representative results are shown. Data are reported as the percent adherence, invasion, or survival of the initial inoculum [e.g., percent survival was calculated as (number of CFU at 60 min/number of CFU at time zero) × 100] normalized to that for the wild type, which was set at 100%. Statistical analysis of the data was performed using one-way analysis of variance (ANOVA) and Student's *t* test.

### Adherence and invasion assays.

Gonococcal adherence and invasion assays were performed with E6/E7 transformed primary cervical epithelial (tCX) cells (79) and cervical carcinoma (ME180) cells (ATCC HTB33), as described previously (58). ME180 and tCX cells were grown as a monolayer in tissue culture coated 96-well plates in MEM (10% FBS) and keratinocyte serum-free medium (K-SFM; supplemented with 0.16 pg/ml recombinant epidermal growth factor [rEGF] and 0.025 mg/ml bovine pituitary extract [BPE] [Gibco]), respectively. N. gonorrhoeae strains were prepared in supplemented MEM or K-SFM and added to cervical cells at an MOI of 10. For adherence assays, the plate was incubated for 1 h at 37°C and then washed three times with PBS. To determine the total number of invasive bacteria, extracellular bacteria were removed by treating the cells with gentamicin (100 μg/ml; Sigma-Aldrich) for 15 min, and the cells were then washed three times with PBS. The total number of adherent and/or invasive bacteria was then determined by lysing the epithelial cells with 0.5% saponin in GC broth and plating serial dilutions.

Infection-blocking assays were performed with ME180 cells as described above, with the following modifications. Bacteria were preincubated with serial dilutions of heat-inactivated MetQ antiserum or preimmune mouse serum (control) in MEM for 30 min at room temperature. The plate was incubated at 37°C with 5% CO_2_ for 10 min to measure adherence.

### Serum survival assays.

Serum was separated from blood taken from healthy volunteers by venipuncture using a Vacuette Z serum separator tube (Greiner Bio-One), in accordance with Griffith University Human Ethics Committee and institutional guidelines. The resistance of N. gonorrhoeae to serum-mediated killing was tested by incubating ∼10^5^ CFU in serial dilutions (10 to 80%) of normal human serum for 1 h at 37°C with 5% CO_2_. Serial dilutions were plated to determine the number of bacterial CFU.

### SBA assays.

The serum bactericidal activity (SBA) of anti-MetQ antibodies was tested by preincubating ∼10^5^ CFU of N. gonorrhoeae in serial dilutions of heat-inactivated anti-MetQ serum or preimmune mouse serum (1:10; control) for 20 min at room temperature, after which normal human serum (preabsorbed with N. gonorrhoeae as described previously [80]) was added to a final concentration of 10% (vol/vol) as a source of complement. The suspension was then incubated at 37°C with 5% CO_2_ for 30 min, and the number of bacterial CFU was determined by plating out serial dilutions.

## Supplementary Material

Supplemental material
