# Supplementary material for: MetQ of Neisseria gonorrhoeae Is a Surface-Expressed Antigen That Elicits Bactericidal and Functional Blocking Antibodies
Source: Infect Immun. 2017 Jan 26;85(2):e00898-16. doi: 10.1128/IAI.00898-16 (PMC5278169; doi:10.1128/IAI.00898-16)
Supplement: Supplemental material [file supp_85_2_e00898-16__index.html]

MetQ of Neisseria gonorrhoeae Is a Surface-Expressed Antigen That Elicits Bactericidal and Functional Blocking Antibodies — Supplemental material 

# MetQ of Neisseria gonorrhoeae Is a Surface-Expressed Antigen That Elicits Bactericidal and Functional Blocking Antibodies

## Supplemental material

- Supplemental file 1 -

  Fig. S1. Coomassie blue-stained SDS-PAGE gel (A) and Western blot of *N. gonorrhoeae* 1291 wild-type (WT), *metQ* knockout (Δ*metQ*), and complemented (C-*metQ*) strains (B).

  PDF, 1.4M
