# Supplementary material for: MetQ of Neisseria gonorrhoeae Is a Surface-Expressed Antigen That Elicits Bactericidal and Functional Blocking Antibodies
Source: Infect Immun. 2017 Jan 26;85(2):e00898-16. doi: 10.1128/IAI.00898-16 (PMC5278169; doi:10.1128/IAI.00898-16)
Supplement: Supplemental material [file IAI.00898-16_zii999091960s1.pdf]

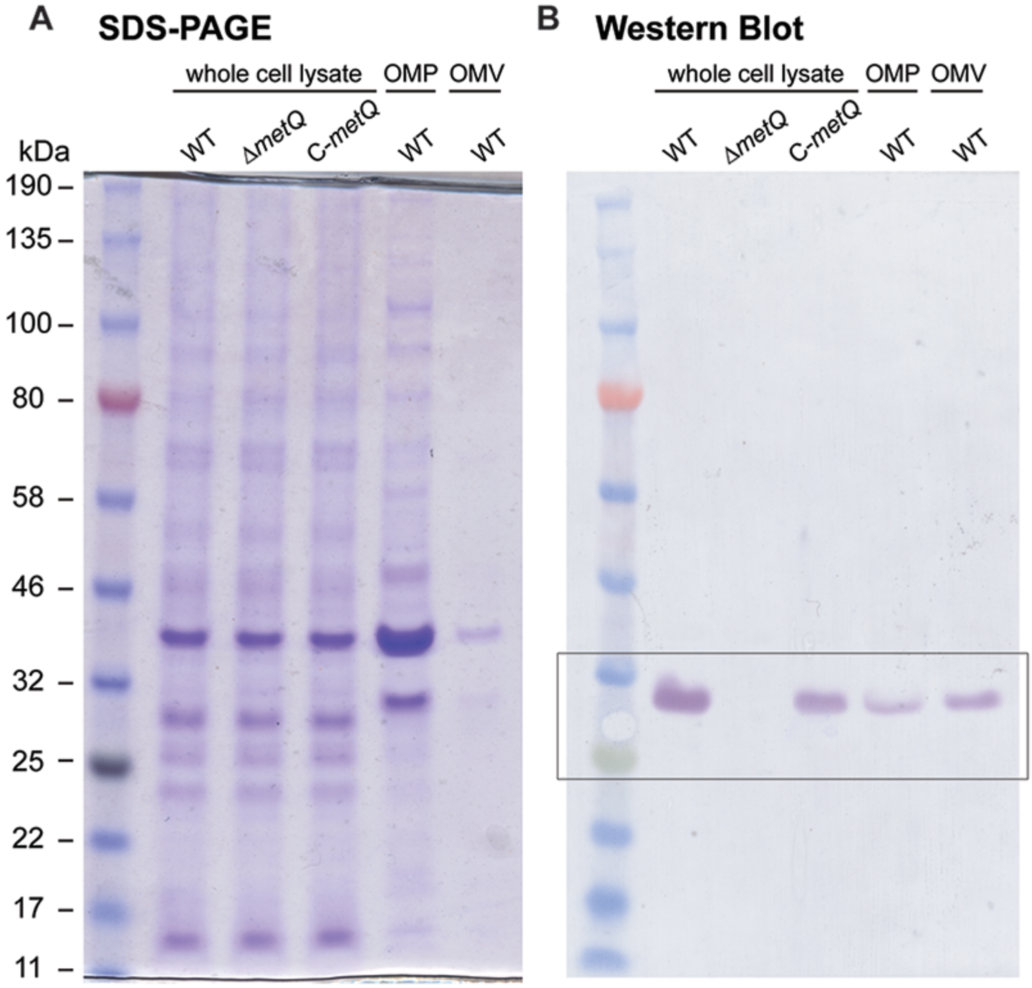

Figure S1. (A) Coomassie stained SDS-PAGE gel and **(B)** Western blot of *N. gonorrhoeae* 1291 wild type (WT), *metQ* knockout (Δ*metQ*) and complemented (C-*metQ*) strains. Samples analyzed include whole cell lysates, outer membrane protein (OMP) fractions, and outer membrane vesicles (OMV). MetQ is detected in the Western blot using polyclonal anti-MetQ antibodies. The boxed region corresponds to the region of the blot shown in Figure 2A.
